# Direct numerical simulation of turbulent dispersion of evaporative aerosol clouds produced by an intense expiratory event

**DOI:** 10.1063/5.0045416

**Published:** 2021-03-31

**Authors:** Alexandre Fabregat, Ferran Gisbert, Anton Vernet, Josep Anton Ferré, Ketan Mittal, Som Dutta, Jordi Pallarès

**Affiliations:** 1 Department d'Enginyeria Mecànica, Universitat Rovira i Virgili, Av. Països Catalans 26, Tarragona 43007, Spain; 2 Sidney Lu Mechanical Engineering Building, University of Illinois at Urbana-Champaign, 1206 W. Green St., MC 244, Urbana, Illinois 61801, USA; 3 Mechanical and Aerospace Engineering, Utah State University, 4130 Old Main Hill, Logan, Utah 84322-4130, USA

## Abstract

Airborne particles are a major route for transmission of COVID-19 and many other infectious diseases. When a person talks, sings, coughs, or sneezes, nasal and throat secretions are spewed into the air. After a short initial fragmentation stage, the expelled material is mostly composed of spherical particles of different sizes. While the dynamics of the largest droplets are dominated by gravitational effects, the smaller aerosol particles, mostly transported by means of hydrodynamic drag, form clouds that can remain afloat for long times. In subsaturated air environments, the dependence of pathogen-laden particle dispersion on their size is complicated due to evaporation of the aqueous fraction. Particle dynamics can significantly change when ambient conditions favor rapid evaporation rates that result in a transition from buoyancy-to-drag dominated dispersion regimes. To investigate the effect of particle size and evaporation on pathogen-laden cloud evolution, a direct numerical simulation of a mild cough was coupled with an evaporative Lagrangian particle advection model. The results suggest that while the dispersion of cough particles in the tails of the size distribution are unlikely to be disrupted by evaporative effects, preferential aerosol diameters (30–40 *μ*m) may exhibit significant increases in the residence time and horizontal range under typical ambient conditions. Using estimations of the viral concentration in the spewed fluid and the number of ejected particles in a typical respiratory event, we obtained a map of viral load per volume of air at the end of the cough and the number of virus copies per inhalation in the emitter vicinity.

## I. INTRODUCTION

Responsible for more than 1.5 × 10^6^ deaths worldwide during 2020, the recent COVID-19 pandemic has made evident the need to better understand how airborne infectious diseases spread among individuals. It is an accepted scientific fact that flow physics plays a central role in the transmission of the SARS-CoV-2 virus (Severe Acute Respiratory Syndrome CoronaVirus), responsible for COVID-19, and other airborne pathogens.^1^ On one hand, the turbulent fluid set in motion by exhalation is responsible for the aerosolization of ejected liquid spewed during a respiratory event.^2–4^ This turbulent flow also controls the dispersion of the associated virus-laden particle cloud in the environment, especially dispersion close to the source of the aerosols. On the other hand, ambient currents produced by ventilation and air conditioning systems dominate the long-term transport^5^ and the surface deposition of the smallest droplets. Finally, occurrence of new infections is also affected by the process of inhaling suspended aerosols in air and the deposition process in the human upper airway.^6^ Thus, understanding how particles are transported by the action of moving fluids is key in designing and implementing preventative strategies including the use of masks,^7–9^ indoor ventilation,^10,11^ and social distancing measures.^12^ Numerical simulations of specific indoor and public transportation environments such as isolation rooms,^13^ airliner cabin sections,^14^ urban buses,^15^ elevators,^16^ parking areas,^17^ restaurants,^18^ and offices^19^ have been conducted in order to design robust transmission mitigation strategies.

Identified as a major pathway for SARS-CoV-2 transmission, the airborne route occurs when pathogen-laden particles are expelled during an expiratory event including talking, singing, coughing, or sneezing. The breakup of the spewed material due to multiple instabilities^20^ generates droplets and aerosols of different sizes.^21–24^ The diameter of the particle and the characteristics of background flow produced by the exhalation are the two most relevant factors controlling the initial residence time and the range of the particle-laden cloud of infectious particles. Historically, particles larger than 100 *μ*m (Refs. 25 and 26) in diameter have been classified as droplets with gravity-dominated dynamics characterized by ballistic trajectories. While these relatively large particles may contain significant amounts of virus copies, their horizontal range and residence time afloat are very limited.^21^ In contrast, the dynamics of particles of 100 *μ*m in diameter or less is dominated by the hydrodynamic drag exerted by the background flow that allows them to remain suspended in the air longer and travel greater distances.

These distinctive transport properties according to each particle size are complicated when the ambient conditions (temperature and humidity) favor the evaporation of the aqueous phase in droplets and aerosols.^7,27^ Most droplets are suspected to evaporate in a few seconds after being released,^28^ forming droplet nuclei consisting mostly of virions, solid residue,^29,30^ and some nonevaporable residual.^31^ With a typical size on the order of 1 *μ*m, each nucleus may potentially remain afloat for hours while carrying multiple virions with an approximate half-life of 1 h.^32^ When a single virion is suspected to potentially cause a new infection,^33^ the importance of this type of droplet nucleus in the transmission of COVID-19 is key. As particles shrink due to evaporative effects, the dominant contribution to their dynamics may transition from gravity to hydrodynamic drag, affecting the particle cloud dispersion.

Using both experiments^34,35^ and numerical simulations,^36,37^ recent studies analyzed several key aspects of the hydrodynamics produced by different expiratory events, including sneezing,^34^ coughing,^36^ talking, and breathing,^35^ and the dispersion processes of the resulting aerosol cloud.

On the numerical front, most studies have relied on some form of the Reynolds Averaged Navier Stokes (RANS) approach to incorporate the impact of turbulence to the overall transport of momentum and scalars, mainly temperature and relative humidity.

Thus, Dbouk and Drikakis used the RANS approach to investigate the effect of background wind currents^36^ and face masks^38^ on the aerosol dispersion of droplets and aerosols produced by expiatory events. Using the RANS methodology for compressible multiphase mixtures and the κ−ω turbulence model for the carrier phase along with a Lagrangian approach for the saliva droplets and aerosols, Dbouk and Drikakis numerically simulated the jet produced by a cough.^36^ They found that an ambient wind speed of 15 km h^−1^ can transport airborne particles to distances of up to 6 m from the source.

The numerical work by Busco *et al.*^34^ used the RANS approach to study the jet flow produced by a sneeze assuming the continuous phase to be a mixture of air and water vapor. Equipped with a κ−ϵ model to account for the turbulent transport contribution and assuming the saliva droplets to be Lagrangian particles, they demonstrated the impact of the exhalation release angle on the airborne droplet cloud dispersion.

The impact of human physiological factors (e.g., illness, stress condition, and anatomy) was addressed by Fontes *et al.* who used numerical simulations to simulate a sneeze.^37^ These authors modeled the carrier phase under the Eulerian framework using Detached Eddy Simulations (DES) with a hybrid solver that combined unsteady Reynolds Averaged Navier–Stokes (URANS) for the flow within the boundary layer and Large Eddy Simulations (LES) in the outer region. Again, droplets were assumed to be Lagrangian particles. Their results suggested that changes in the nasal and buccal passages may have a significant impact on the horizontal range traveled by the ejected droplets with nasal obstruction, potentially leading to increases of up to 60%. In addition, physico-chemical characteristics of the saliva were found to affect the overall dispersion properties of the spray generated by a sneeze.

Using LES, Pendar and Pascoa studied the dispersion of saliva droplets produced by violent air exhalation events^39^ such as coughing and sneezing. Similar to Fontes *et al.*, they used an Eulerian approach for the carrier phase assuming respiratory droplets to obey the Lagrangian dynamics. Assuming a compressible flow, the hydrodynamics was modeled using LES with a subgrid-scale stress model based on a one equation eddy-viscosity model. The authors found that for a strong sneeze, large droplets of 540 *μ*m in diameter could travel up to 4 m with droplets taking up to 3 s to settle down in the absence of background flow. They observed that bending of the head during sneezing could reduce the droplet travel distance by 22% and that wearing a face mask could further reduce the droplet travel distance to 0.6 m. In addition, they also concluded that background flow turbulence must play a key role in the flow physics of jets produced by expiratory events, stressing the need for accurate modeling of the fluctuating transport contribution to the flow dynamics.

Alternative approaches to turbulent modeling based on random walk methodology were used by Wang *et al.* to determine the effect of several environmental factors on droplet transport and dispersion.^40^ Renzi and Clarke showed that the dynamics of the jet produced by expiratory events could be modeled by extending the theory of buoyant vortex rings.^41^ By coupling the integral model for a continuous phase and the Lagrangian particle-tracking approach, these authors explored the effect of initial conditions on the horizontal range of the droplet cloud. The reported key role of the vortex structure on keeping droplets afloat and increasing the residence time of airborne particles provides additional motivation to study in detail the turbulent structure of the flow produced by the rapid air exhalations produced by coughs.

In order to gain insight into the role played by the particle size and the evaporation on the dispersion of particle clouds produced by a mild violent expiratory event, we numerically simulated the trajectories of thousands of particles evolving in the flow produced by an idealized cough solved using Direct Numerical Simulations (DNS). This simulated cough can be divided into two stages: during the exhalation that lasts for 0.4 s, air generates a warm and moist jet that horizontally penetrates into an initially quiescent environment at lower temperature and water vapor content. Once the exhalation ceases, the jet evolves into a thermal puff that continues to grow as it bends in the vertical direction by the action of buoyancy and dissipates due to viscous effects. By considering both evaporable and nonevaporable particles with seven different initial diameters, ranging from 4 to 256 *μ*m, we could numerically investigate with an unprecedented detail the evolution of respiratory particles traveling in the air under typical temperature and relative humidity conditions.

From the point of view of fluid dynamics, respiratory events generate multiphase jets with more or less rich dynamics. The specific length and spatial scales of the problem determine the flow regime and the distribution of exhalation velocity and particle sizes at release.^42^ The flow realization presented here lasts from the onset of the cough at *t* = 0 until *t* = 1.65 s. This time span covers the initial dispersion regime over which particle dispersion is dominated by the hydrodynamic drag of the jet-to-plume flow up to approximately t≈0.75 s. At that point, the average speed of the particle cloud has dropped to typical indoor air speed levels at which time ambient air currents would take over the dispersion of the aerosols. By assuming the reasonable values of the viral concentration in the spewed material, the aerosol concentration field obtained from the fully resolved simulation allows us to estimate the spatial distribution of the COVID-19 infection risk at the last stages of a mild cough under typical ambient conditions.

In the following sections, first, in Sec. II, the numerical model has been described in detail, covering both governing equations and the pertinent boundary conditions for both the continuous and dispersed phases. In Sec. III, the results have been discussed, illustrating the effect of evaporation on aerosol dispersion. Finally, the paper concludes with summarizing the main findings and listing future research directions.

## II. PHYSICAL AND MATHEMATICAL MODELS

The flow produced by a violent expiratory event was investigated by Fabregat *et al.*^43^ who used DNS to numerically simulate an idealized, isolated mild cough occurring in an isothermal and stagnant environment. In the flow setup, exhaled air, with the temperature and relative humidity of $T_{0}=34$ °C and $\xi_{0}=85$%, was injected through a cylindrical pipe of diameter *d* representing the mouth into a quiescent environment at $T_{\infty }=15$ °C and $\xi_{\infty }=65$%. To mimic the transient nature of the violent exhalation, the inlet velocity *w*_0_ was assumed to linearly increase from $w_{0}(t=0)=0$ to the peak value $w_{m}=4.8$ m s^−1^ at $t_{m}=0.15$ s to then linearly decayed back to zero at $t_{c}=0.4$ s, i.e.,

$$w_{0}(t)=\left\{\begin{array}{cc} \frac{w_{m}}{t_{m}}t & 0\leq t<t_{m}\\ w_{m}-\frac{w_{m}}{t_{c}-t_{m}}\left(t-t_{m}\right) & t_{m}\leq t\leq t_{c}\\ 0 & t>t_{c}. \end{array}\right.$$ (1)

This velocity sequence reproduces the overall characteristics of a cough in agreement with the measurements reported by Gupta *et al.*^42^ As the exhaled moist air accelerated/decelerated according to Eq. (1), it produced a jet that penetrated into the unperturbed ambient air. Once the cough ceased for $t>t_{c}$, the jet evolved into a buoyant puff characterized by the deflection of the front trajectory due to buoyancy and the decay of turbulence due to dissipative effects.

**FIG. 1.**
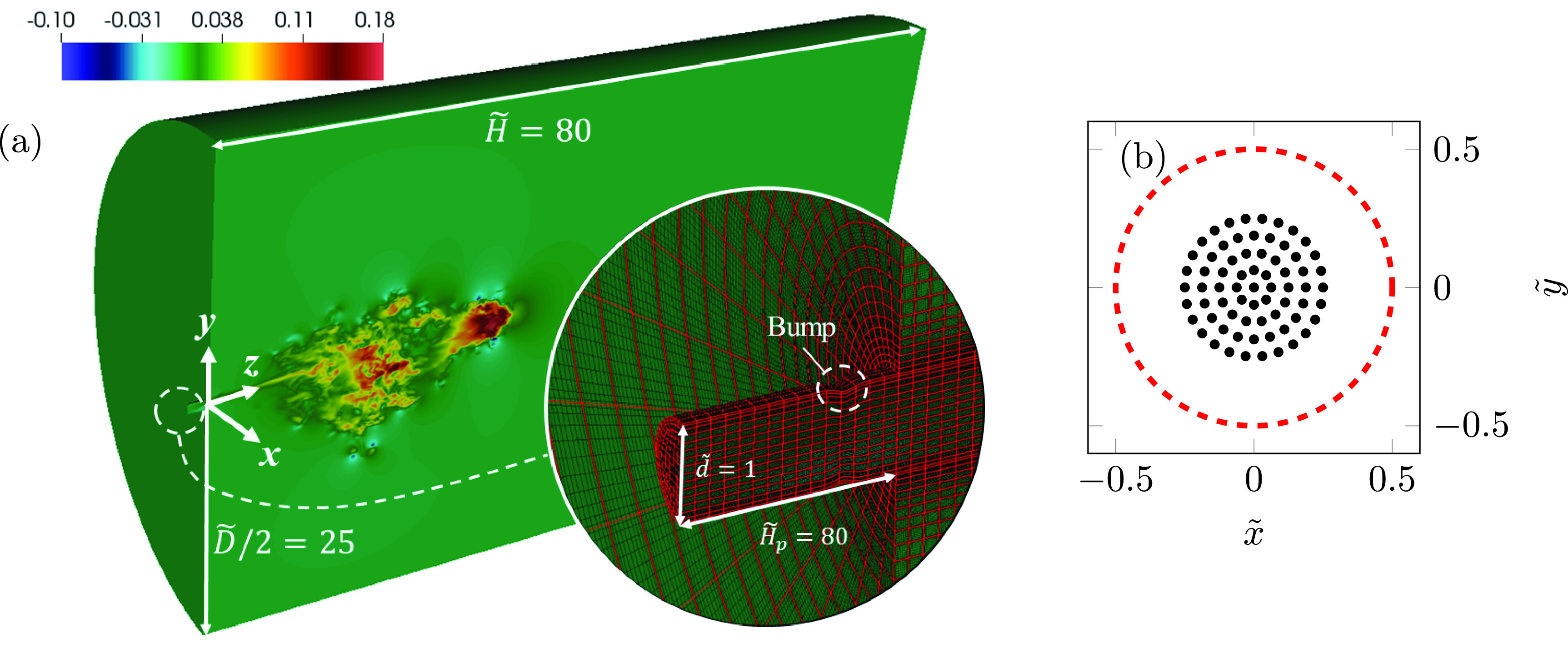
(a) A sliced domain showing an instantaneous $\tilde{w}$ velocity component field. The inset shows details of the inlet section and the computational mesh with spectral element boundaries in red and the polynomial expansion Legendre–Gauss–Lobatto collocation points in black.^43^ (b) Location of the *n_l_* = 69 particle seeding positions (in black) with respect to the inlet cross section (red dashed line).

Assuming flow incompressibility and using the inlet diameter *d*, the peak cough velocity *w_m_* and the temperature difference $\Delta T=T_{0}-T_{\infty }$ as length, velocity, and temperature scales, the nondimensional mass, momentum, and energy conservation equations can be written as

$$\frac{\partial {\tilde{u}}_{i}}{\partial {\tilde{x}}_{i}}=0,$$ (2)

$$\frac{\partial {\tilde{u}}_{i}}{\partial \tilde{t}}+{\tilde{u}}_{j}\frac{\partial {\tilde{u}}_{i}}{\partial {\tilde{x}}_{j}}=-\frac{\partial \tilde{p}}{\partial {\tilde{x}}_{i}}+\frac{1}{Re}\frac{\partial^{2}{\tilde{u}}_{i}}{\partial {\tilde{x}}_{j}\partial {\tilde{x}}_{j}}+Ri\;\tilde{\theta }\;\delta_{i2},$$ (3)

$$\frac{\partial \tilde{\theta }}{\partial \tilde{t}}+{\tilde{u}}_{j}\frac{\partial \tilde{\theta }}{\partial {\tilde{x}}_{j}}=\frac{1}{Pe}\frac{\partial^{2}\tilde{\theta }}{\partial {\tilde{x}}_{j}\partial {\tilde{x}}_{j}},$$ (4)

where $\tilde{t}=tw_{m}/d$ is the time, $\tilde{p}=p/\rho w_{m}^{2}$ is the pressure, $\tilde{\theta }=(T-T_{\infty })/\Delta T$ is the nondimensional temperature perturbation, *δ_ij_* is the Kronecker delta, and ${\tilde{u}}_{i}=(\tilde{u},\tilde{v},\tilde{w})$ is the velocity field with coordinates ${\tilde{x}}_{i}=(\tilde{x},\tilde{y},\tilde{z})$ in the spanwise, gravity-aligned, and streamwise directions. Note that the tilde symbol is reserved for nondimensional variables. The air thermal conductivity, kinematic viscosity, thermal diffusion coefficient, and thermal expansion coefficient evaluated at $T^{*}=(T_{0}+T_{\infty })/2$ were assumed to remain constant at $k_{a}=0.026$ W m^−1^ K^−1^, $\nu_{a}=1.6\times 10^{-5}$ m^2^ s^−1^, $\alpha_{a}=2.24\times 10^{-5}$ m^2^ s^−1^, and $\beta_{a}=0.00347$ K^−1^, respectively. Density variations with temperature were considered only in the buoyancy term of the vertical momentum equation according to the Boussinesq approximation [with $\rho_{a}=\rho (T^{*})=1.22$ kg m^−3^]. The Reynolds, Richardson, and Péclet numbers were $Re=w_{m}d/\nu_{a}=6000$, $Ri=g\beta_{a}\Delta Td/w_{m}{}^{2}=5.61\times 10^{-4}$, and $Pe=w_{m}d/\alpha_{a}=4200$, respectively. The gravity acceleration was $g\;\delta_{i2}=-9.8$ m s^−2^. The flow was integrated up to t≈1.7 s. The hydrodynamics is illustrated in Fig. 1(a), which shows a slice of the instantaneous *w* field at *t* = 0.75 s. This panel also shows the computational domain dimensions and some details of the cylindrical inlet and numerical mesh.

High-Order Spectral Element Methods (HO-SEMs) have been shown to be especially well suited for turbulent flows.^44^ The Nek5000 HO-SEM solver^45^ is used here to solve Eqs. (2)–(4) in the velocity–pressure form using the semi-implicit *k*th-order backward difference formula (BDF*k*)/*k*th-order extrapolation (EXT*k*) time-stepping in which the time derivative is approximated by a *k*th-order backward difference formula (BDF*k*), the nonlinear terms (and other forcing) are treated with a *k*th-order extrapolation (EXT*k*), and the viscous and pressure terms are treated implicitly (in this work, we used *k* = 3). This approach leads to a linear unsteady Stokes problem to be solved at each time step, which comprises a Helmholtz equation for each component of velocity (and temperature/scalar) and a Poisson equation for pressure (see, e.g., Sec. 2.2 of Mittal *et al.*^46^). The numerical simulation presented here, with approximately 370 × 10^9^ mesh points, used 20 Intel Platinum 8168 nodes with 24 cores each interconnected with a 100 Gb/s Infiniband network. The average CPU time per time step is around 5 s. The simulation took $5.19\times 10^{5}$ CPU hours.

Laboratory measurements used to estimate the production of turbulent kinetic energy in self-similar momentum puffs by Glezer and Coles.^47^ suggested values around $\Pi =100{\left(I/\rho_{f}\;t^{5}\right)}^{1/2}$ within the vortex ring, where *I* is the puff impulse ($I=6\times 10^{-4}$ N s in the present DNS study). Under the assumption of equal production and dissipation, the ratio between the Kolmogorov length scale *η_K_* and the exit diameter can be estimated as $\eta_{K}/d\approx 0.01\;t^{5/8}$. This result is compatible with estimations from DNSs of jets based on the measurements of Panchapakesan and Lumley^48^ (see, for example, Boersma *et al.*^49^). For the present Reynolds number based on the maximum velocity during the cough, $\eta_{K}/d\approx 6\times 10^{-4}x/d$. The present simulation shows that the initially laminar jet becomes completely turbulent, with fine scale activity, at *t* = 0.3 s ($\tilde{t}=72$). At this time, the puff is approximately located at *z* = 15 ($\tilde{z}=15d$) with values of the nondimensional Kolmogorov length scale of approximately $5\times 10^{-3}$ and $10^{-2}$ according to the criteria based on the measurements of Glezer and Coles^47^ and Panchapakesan and Lumley,^48^ respectively. The corresponding grid sizes at the jet axis at this position are $\Delta \tilde{x}=\Delta \tilde{y}\approx 0.009$ and $\Delta \tilde{z}\approx 0.04$, which are of the same order of magnitude as the estimations. A detailed discussion on the cough hydrodynamics can be found in Fabregat *et al.*^43^

### A. Dispersed phase model

Given the relatively initial small particle volume fraction (approximately $10^{-5}$) in real coughs and sneezes,^50^ it is assumed that the particles do not affect the flow (one-way coupling). Under this hypothesis, the solver for the carrier phase hydrodynamics was coupled with a particle advection model to obtain the trajectories $s_{j}({\vec{x}}_{j},t)$ of $j=1,\ldots ,N_{p}$ particles that represent the ejecta fluid. Although, immediately after being spewed, the ejected material typically undergoes filamentation and a breakup process due to capillary instabilities, the dispersed phase rapidly evolves into droplets and aerosols of spherical shape with sizes, typically ranging between 1 and several hundred *μ*m in diameter.^50^ Mostly composed of water,^51^ the dispersed phase density and thermal conductivity are set to $\rho_{p}=1000$ kg m^−3^ and $k_{p}=0.606$ W m^−1^ K^−1^, respectively.

Particles are released using *n_b_* = 200 batches equally spaced in time over approximately the entire duration of the cough (i.e., one batch released every 2 ms). To investigate the effect of droplet size and evaporation, particles of *n_s_* = 7 different initial diameters (4, 8, 16, 32, 64, 128, and 256 *μ*m), both evaporating and nonevaporating (*n_e_* = 2), are released from *n_l_* = 69 fixed seeding positions, resulting in a total number of particles $N_{p}=n_{b}n_{s}n_{e}n_{l}=193\;200$ by the end of the expiratory event. The sizes of the particles were selected from the typical ranges reported for coughs.^50^ According to the estimations of the Kolmogorov length scale indicated above ($\eta_{K}\approx 2\times 10^{-4}\;m$), only the largest particles of 256 *μ*m, which are dominated by the gravitational settling, are larger than this length scale. The nondimensional location of the *n_l_* particle seeding positions is shown in Fig. 1(b). The particles are seeded over a circular area centered at $\left(\tilde{x},\tilde{\;y},\;\;\tilde{z})=(0,\;0,\;0\right)$. Depositions on the inlet vicinity boundaries are prevented by using an outer diameter for the seeding area equal to half the inlet diameter [red dashed line in Fig. 1(b)].

Due to the presence of the nonevaporable material in the ejected fluid mostly composed of proteins and salts, a lower bound for the particle diameter representing the size of the nuclei^52^ has been set to $0.3d_{p}^{0}$, where $d_{p}^{0}$ represents the initial particle size. Thus, the fraction of remaining evaporable water *ς* in a particle can be defined as

$$\varsigma =1-\frac{10}{7}\frac{\left(d_{p}^{0}-d_{p}\right)}{d_{p}^{0}},$$ (5)

where *ς* ranges between 1 at release time and 0 when no evaporable water is left. Due to the modest change in particle density as water evaporates, *ρ_p_* has been assumed constant over the duration of the numerical experiment.

The position of an idealized spherical and smooth particle *X_i_* can be written as

$$\frac{d{\tilde{X}}_{i}}{d\tilde{t}}={\tilde{U}}_{i},$$ (6)

where the particle velocity *U_i_* can be obtained by solving the balance between hydrodynamic drag, buoyancy, and thermophoresis forces,

$$\frac{d{\tilde{U}}_{i}}{d\tilde{t}}=\underset{\text{Drag}}{\underset{︸}{\frac{{\tilde{u}}_{i}-{\tilde{U}}_{i}}{\tau_{p}}}}+\underset{\text{Buoyancy}}{\underset{︸}{n_{g}\delta_{i2}}}+\underset{\text{Thermophoresis}}{\underset{︸}{n_{th}\frac{\partial \tilde{\theta }}{\partial {\tilde{x}}_{i}}.}}$$ (7)

The lift force, the pressure gradient force, and the virtual mass and Basset forces are neglected according to the relatively small density ratio between the fluid and the particle. The hydrodynamic drag accounts for the fluid resistance or friction a particle experiences as it moves with respect to the carrier phase and can be characterized by the particle Stokes number *τ_p_* defined as the ratio of the particle and flow characteristic times. Small Stokes numbers are associated with particles that rapidly react to changes in accelerations in the underlying flow, while large Stokes are found in inertia-dominated particles. The Stokes number for a particle of nondimensional diameter ${\tilde{d}}_{p}$ can be written as

$$\tau_{p}=Re\frac{{\tilde{d}}_{p}^{2}}{18C_{c}}\frac{\rho_{p}}{\rho_{a}}{\left(1+0.15Re_{p}^{0.687}\right)}^{-1},$$ (8)

where the last term is a correction of the Stokes drag when the particle Reynolds number $Re_{p}=d_{p}||u_{i}-U_{i}||/\nu_{a}$ does not remain small.^53^ In this equation, *C_c_* is a correction factor that depends on the Knudsen number *Kn* defined as

$$Kn=\frac{2\lambda }{d_{p}},$$ (9)

$$C_{c}=1+Kn\left[1.205e^{-0.0026/Kn}+0.425e^{-0.7400/Kn}\right],$$ (10)

where $\lambda =6.8\times 10^{-8}$ m is the free mean path for air at $T^{*}$.^54^

The magnitude of the buoyancy force experienced by a particle floating in a fluid with different densities under a gravitational field with acceleration magnitude *g* can be written as

$$n_{g}=-g\left(1-\frac{\rho_{a}}{\rho_{p}}\right)\frac{d}{w_{m}^{2}}.$$ (11)

The thermophoretic force arises because of gradients in the temperature field across the particle characteristic length, *d_p_*, for a spherical particle.^53^ The prefactor of the nondimensional temperature gradient, *n_th_*, is defined as

$$n_{th}=-\frac{18}{Re^{2}}\frac{K_{tp}}{{\tilde{d}}_{p}^{2}}\frac{\Delta T}{T^{*}}\frac{\rho_{f}}{\rho_{p}},$$ (12)

where the factor *K_tp_*, which is defined as^55^

$$K_{tp}=\frac{2C_{s}\left(k_{f}+2k_{p}Kn\right)\left[1+2Kn\left(1.2+0.41\;\text{exp}\;(-0.44/Kn)\right)\right]}{\left(1+6C_{m}Kn\right)\left(2k_{f}+k_{p}+4k_{p}C_{t}Kn\right)},$$ (13)

contains three constants set to $C_{s}=1.17,\;C_{m}=1.14$, and $C_{t}=2.18$.

Note that the negative sign in *n_th_* indicates that the force points in the direction of maximal temperature decay.

After solving for the carrier phase velocity ${\tilde{u}}_{i}$ by integrating in time the hydrodynamics [Eqs. (2)–(4)], the dispersed phase [Eqs. (6) and (7)] is temporally integrated using the Exponential-Lagrangian Tracking Scheme (ELTS) derived by Barton.^56^ While classical explicit methods exhibit prohibitive computational costs due to numerical instability constraints for relatively small values of *τ_p_* (e.g., $\Delta t<2\tau_{p}$ for fourth-order Runge–Kutta methods), the ELTS is inherently stable and allows us to use the same time step for both the carrier and disperse phases.

### B. Evaporation model

The nondimensional equation for the rate of change in particle size due to evaporation of the aqueous fraction, which can be obtained from the unsteady mass balance of the particle,^57^ can be written as

$$\frac{d\;{\tilde{d}}_{p}}{d\;\tilde{t}}=\frac{4}{Re\;Sc_{v}}\frac{\rho_{f}-\rho_{s}}{\rho_{p}}\frac{1}{{\tilde{d}}_{p}},$$ (14)

where *ρ_s_* is the saturation water vapor concentration at the droplet surface, *ρ_f_* is the local ambient water vapor concentration, *D_v_* is the water vapor diffusivity in air, and $Sc_{v}=\frac{\nu_{a}}{D_{v}}$ is the Schmidt number for water vapor.

The evolution of the particle temperature, derived from the thermal energy balance of the particle,^57^ can be written as

$$\frac{d\;{\tilde{\theta }}_{p}}{d\;\tilde{t}}=\frac{12}{Re\;Pr_{p}}\frac{1}{{\tilde{d}}_{p}^{2}}\left[\frac{k_{v}}{k_{p}}\left({\tilde{\theta }}_{f}-{\tilde{\theta }}_{p}\right)+\frac{D_{v}\Delta H_{v}}{k_{p}\Delta T}\left(\rho_{f}-\rho_{s}\right)\right],$$ (15)

where $\nu_{p}=1.015\times 10^{-6}$ m^2^ s^−1^ is the water kinematic viscosity at $T^{*}$, *θ_p_* and *θ_f_* are the particle and ambient fluid nondimensional temperatures, $Cp_{p}=4179.6$ J kg^−1^ K^−1^ is the liquid water heat capacity, $k_{v}=0.026$ W m^−1^ K^−1^ and $k_{p}=0.606$ W m^−1^ K^−1^ are the water vapor and liquid water thermal conductivities, $\alpha_{p}=k_{p}/(\rho_{p}Cp_{p})=1.45\times 10^{-7}$ m^2^ s^−1^ is the liquid water thermal diffusivity, $\Delta H_{v}=2.257\times 10^{6}$ J kg^−1^ is the water enthalpy of vaporization, and $Pr_{p}=\nu_{p}/\alpha_{p}=7.0$ is the dispersed phase Prandtl number. Equation (14) assumes that the Sherwood and Nusselt numbers are constant (Sh=Nu=2)^53^ given the small particle Reynolds numbers involved. We found that the use of a higher Reynolds correlation does not influence significantly the results. Specifically, for the largest particles considered, of 256 *μ*m, falling freely from a height of 1.5 m, the differences of the diameter using Sh=Nu=2 or the correlation proposed by Ranz and Marshall^58^ when they reach the floor are less than 1%.

The saturation water vapor concentration *ρ_s_* is determined, using the ideal gas law, as

$$\rho_{s}=\frac{p_{s}(T_{p})}{RT_{p}},$$ (16)

where the water vapor pressure *p_s_* in Pa is computed from the correlation^59^

$$p_{s}(T)=\exp(-5.800\;220\;6\times 10^{3}/T+1.391\;499\;3-\;4.864\;023\;9\times 10^{-2}\;T+4.176\;476\;8\times 10^{-5}\;T^{2}-\;1.445\;209\;3\times 10^{-8}{\;T}^{3}+6.545\;967\;3\log T),$$ (17)

where *T* is in K. Recall that $T=T_{\infty }+\Delta T\tilde{\theta }$.

The local ambient water vapor concentration *ρ_f_* is determined as

$$\rho_{f}=\frac{p_{s}(T_{f})}{RT_{f}}\frac{\xi }{100},$$ (18)

where the relative humidity *ξ* can, then, be calculated using^59^

$$\xi =\frac{p_{0}}{p_{s}}\left[\frac{x_{v}}{0.621\;98+0.378\;02x_{v}}\right],$$ (19)

with the total pressure taken to be $p_{0}=101\;325$ Pa. Since both heat and water vapor transport equations are only distinguished by their Peclet number and given the similitude between the Prandtl and Schmidt numbers (*Pr* = 0.7 and *Sc* = 0.6, respectively), differences in the transport between both scalars due to diffusive effects have been neglected and the temperature field has been used as a proxy for the water content in the air. Thus, the mass fraction of water in the air *x_v_* can be computed assuming a linear relationship between temperature and the mass fraction of water vapor (i.e., for $T=T_{\infty }=15$ °C or $\tilde{\theta }=0,\;x_{v}=0.007$ and for $T=T_{0}=34$ °C or $\tilde{\theta }=1,\;x_{v}=0.0273$),

$$x_{v}=0.007+0.0203\;\tilde{\theta }.$$ (20)

Given the relatively small particle volume fraction, this model neglects the heat transfer from the dispersed to the carrier phase.

At each time step, the disperse phase transport equations [(6) and (7)] are integrated in time with the updated values of particle diameter and particle temperature obtained by integrating in time the ordinary differential equations [(14) and (15)] with the same BDF3 scheme used to integrate the hydrodynamics.

## III. RESULTS

The simulated expiratory event allows us to investigate the role of initial particle size and evaporation on the resulting aerosol cloud dispersion under typical temperature and relative humidity conditions. Inspection of the relevance of each force in Eq. (7) demonstrates that the contribution due to thermophoresis is negligible in comparison to hydrodynamic drag and buoyancy terms.

**FIG. 2.**
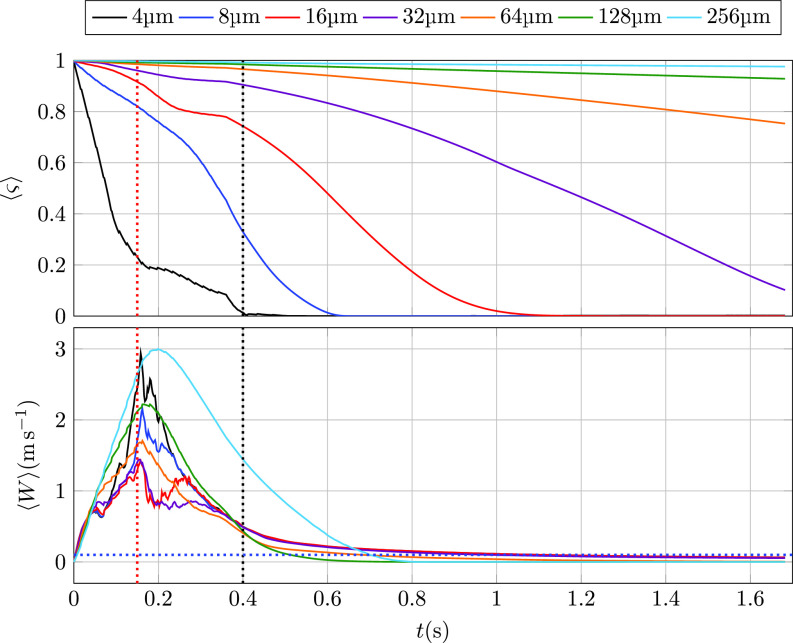
Top: cloud-averaged fraction of remaining evaporable water *ς* for each particle size. Bottom: cloud-averaged vertical velocity component (*W*) for each size of evaporative particles. Red and black vertical dashed lines indicate *t_m_* and *t_c_*, respectively. The blue horizontal dashed line indicates a typical indoor mean air velocity of 0.1 ms^−1^.

Temporal evolution of instantaneous averages across cloud particles, represented by ⟨·⟩, for the fraction of remaining evaporable water *ς* defined in Eq. (5) and the streamwise velocity *W* are shown in the top and bottom panels of Fig. 2, respectively. The time to complete evaporation is found to strongly depend on the initial particle size. By the end of the experiment, particles of initial diameter 16 *μ*m and smaller have completely evaporated, while droplets of 64 *μ*m and larger still retain more than 75% of the free water at release.

As shown in the bottom panel of Fig. 2, the horizontal speed of the cloud peaks at approximately *t_m_* (red dashed vertical line) when exhalation reaches its maximum velocity. By the end of the numerical experiment, the cloud has lost most of its momentum with the values of the averaged vertical velocity component (⟨W⟩) around 0.05 m s^−1^. This result suggests that while particle transport is initially controlled by the flow induced by the expiratory event, once the momentum dissipates and the cloud velocity declines to typical indoor conditions^60^ with an average velocity of about 0.1 ms^−1^, the dispersion processes, thereafter, will most likely be dominated by the ambient air currents.

**FIG. 3.**
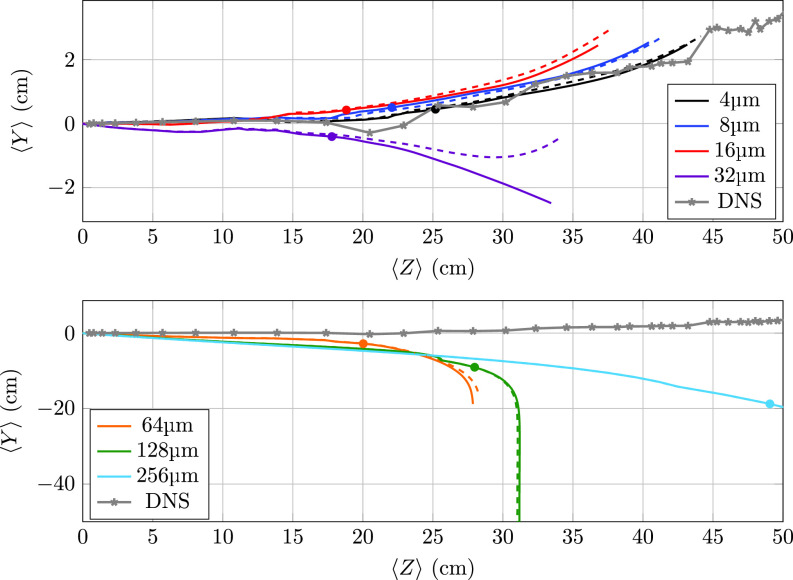
Trajectory of particle cloud centroid for evaporative (dashed) and nonevaporative (solid) types. Markers indicate the cough ceasing time for the nonevaporating type. Top: 4, 8, 16, and 32 *μ*m. Bottom: 64, 128, and 256 *μ*m.

The temporal evolution of the cloud centroid $\left\langle X_{i}\right\rangle$ for each particle size and evaporation mode is shown in Fig. 3. The top and bottom panels show the results for the drag-dominated (4–32 *μ*m) and gravity-dominated (64–256 *μ*m) particles, respectively. Each symbol represents the cough end time *t_c_* for each nonevaporative case (for the sake of clarity, the very similar cough end time for the evaporative case is not shown).

The small differences between the cloud centroid trajectory for evaporating (dashed lines) and nonevaporating (solid lines) particles under 32 *μ*m in diameter suggest that evaporation has a negligible impact on the aerosol dispersion. The drag-dominated transport of these aerosols exerted by the background flow can only intensify as the particle diameter further decreases due to evaporation. Notably, over the duration of the numerical experiment, both the 128 and 256 *μ*m particles have mostly reached the boundaries of the domain located at $r=\tilde{R}=25$ (see Fig. 1). The largest ones, with larger inertia, are the particles that travel the farthest before reaching to bottom domain limits.

Conversely of the particle distribution, particles with a diameter above 32 *μ*m also exhibit negligible differences in their cloud centroid trajectory. In this case, and as shown in Fig. 2, the shrinking in size due to evaporation over the duration of the experiment is too small to significantly change the particle dynamics dominated by the gravitational action.

Notably, evaporation is found to significantly modify the path followed by the 32 *μ*m cloud. While nonevaporative particles follow a quasi-parabolic trajectory similar to that described by larger particles, the evaporative cloud counterpart reverses its downward trajectory. This behavior suggests that for this specific size, evaporation leads to a transition from gravity to drag dominated dispersion.

To compare both phases, the trajectory followed by the frontal region of the thermal puff produced by the cough^43^ is included in Fig. 3. As expected, as the particle size decreases, the transport contribution due to hydrodynamic drag increases, and both the puff front and particle cloud paths tend to overlap. As gravitational effects increase for particles above 16 *μ*m, the trajectory departure from the rising thermal puff strengthens.

**FIG. 4.**
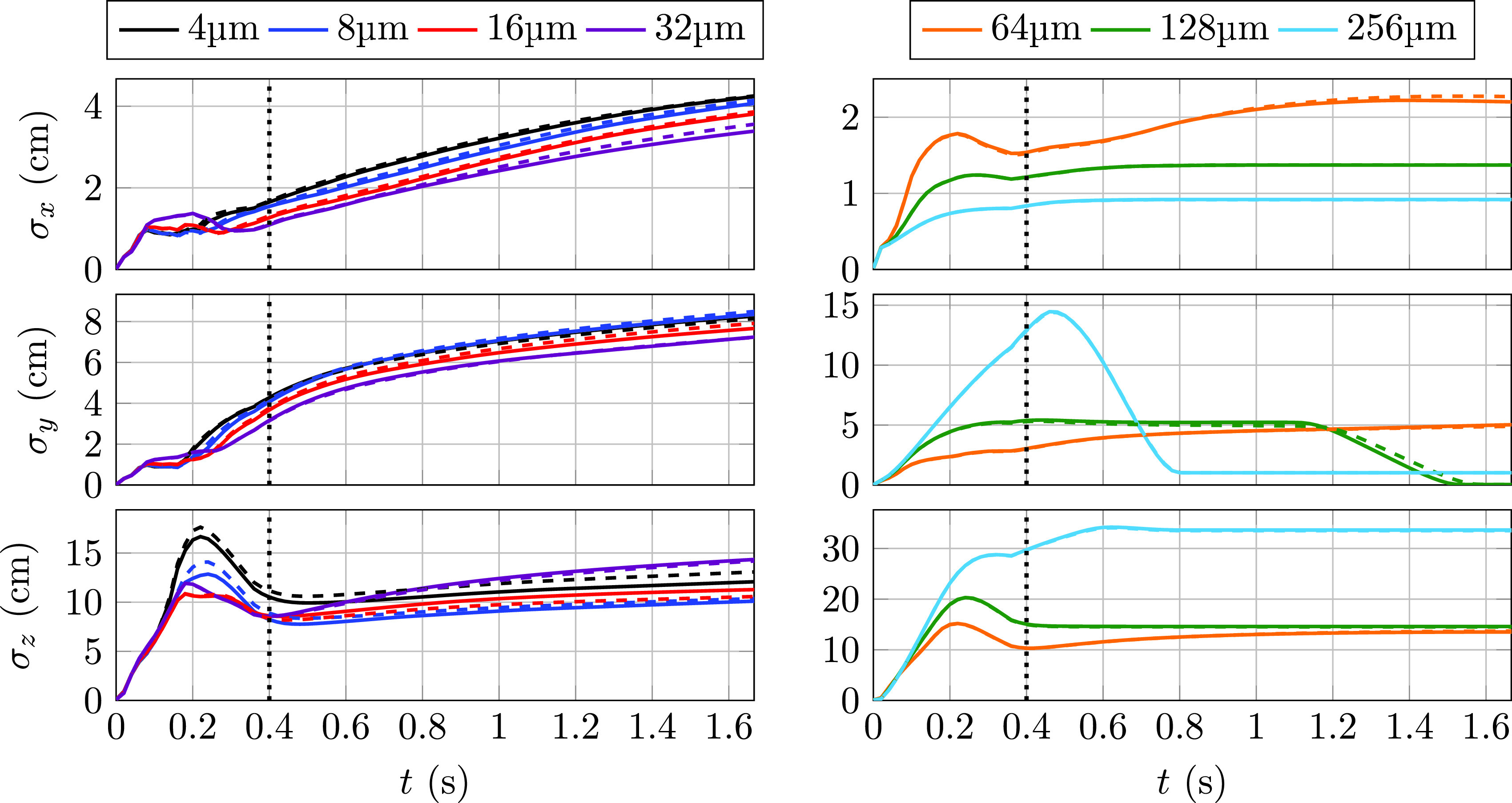
Temporal evolution of each size cloud variance in each direction for evaporative (dashed) and nonevaporative (solid) types. Left: 4, 8, 16, and 32 *μ*m. Right: 64, 128, and 256 *μ*m. The black dotted line marks the time the cough ceases.

Figure 4 shows the cloud size, estimated from the standard deviation of the particle positions with respect to the centroid *σ_i_*,

$$\sigma_{i}(t)=\frac{\sum_{j=1}^{N_{p}}\sqrt{{\left(X_{i,j}(t)-\langle X_{i}\rangle \right)}^{2}}}{N_{p}},$$ (21)

for particles of 4–32 *μ*m (left panels) and 64–256 *μ*m (right panels) in diameter for both evaporating (dashed) and nonevaporating (solid) modes. The landing of most of the 128 and 256 *μ*m particles on the computational domain boundary, where they remain deposited eventually, leads to constant values of *σ_i_* in all directions. The start and end times of this deposition process roughly span from the peak to the nearly zero values of *σ_y_* for these two particle sizes.

Estimated from *σ_x_*, the spanwise width inversely grows with the particle size for the 4–32 *μ*m range with very similar values in *σ_y_* and *σ_z_*.

**FIG. 5.**
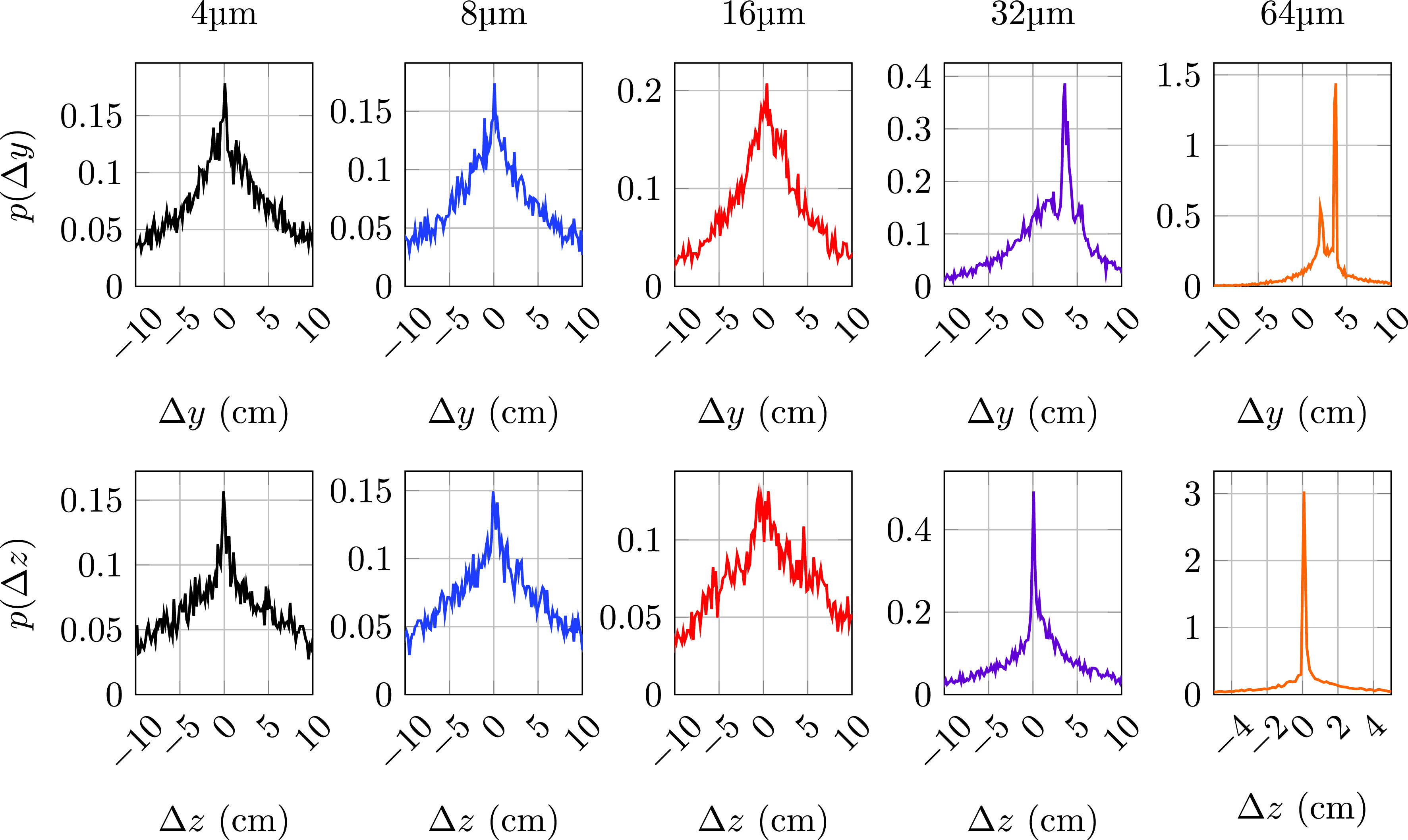
Probability density distribution (PDF) of the position difference in *y* (top row) and *z* (bottom row) between evaporative and nonevaporative particles for each size at t≈1.68 s. The two largest particles are not shown due to very small differences.

To gain insight into the effects of evaporation and estimate the impact of the droplet size decrease on the particle dispersion, Fig. 5 shows the probability density function of the particle position difference between the evaporative and nonevaporative cases at the latest simulation time. The results for the distribution of vertical positions (top row of Fig. 5) suggest that evaporation leads to significant rises in *y* only for release diameters above 16 *μ*m with values above 64 *μ*m (not shown here for simplicity) being negligible. The results indicate that there is a significant skewness value in the vertical position difference distribution for 32 and 64 *μ*m particles with a mean vertical rise of approximately 4 cm for the evaporative case.

The distribution of the *z* position difference (bottom row of Fig. 5) suggests that the impact on the horizontal range for particles smaller than 32 *μ*m and larger than 64 *μ*m (not shown) in diameter is negligible. The distribution for 32 and 64 *μ*m, although slightly skewed toward positive values, still exhibits zero average.

**FIG. 6.**
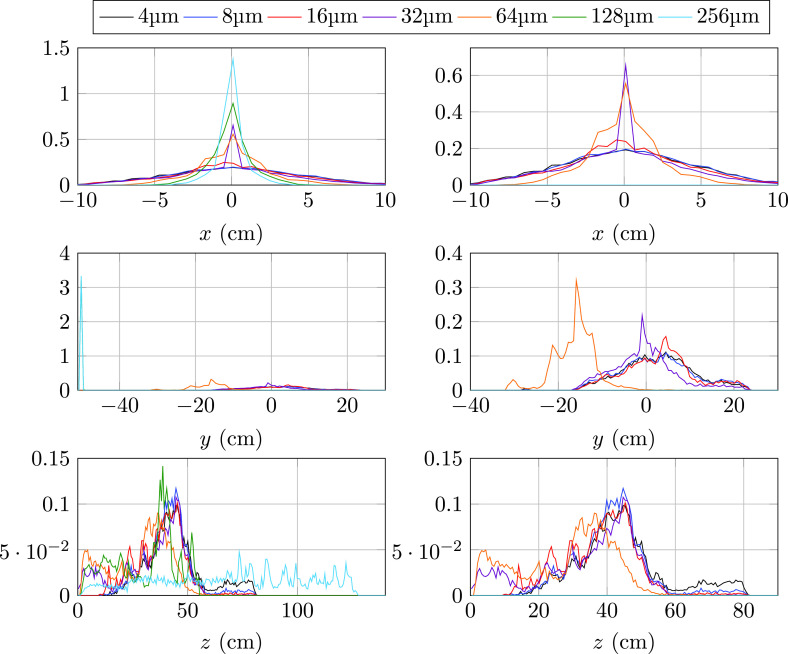
Probability density distribution (PDF) of the terminal spatial location in each coordinate for evaporative particles. Left: all sizes. Right: 4–64 *μ*m.

Particle position distribution in each spatial direction for the evaporative case at the end of the numerical experiment is shown in Fig. 6 for all particle sizes (left panels) and only the five smaller diameters (right panels). As indicated by the standard deviation results in Fig. 4, lateral cloud spreading increases as the particle diameter decreases. Differences in the vertical position distributions are negligible between particles from 4 to 16 *μ*m. As the particle diameter increases, the cloud falls due to gravity intensify with 64 *μ*m droplets still afloat and with 128 and 256 *μ*m droplets completely landed on the domain boundaries. The largest particles, capable of retaining more injection momentum, exhibit the largest horizontal reach up to approximately 125 cm from the source before landing. Interestingly, the 4–16 *μ*m particles, capable of staying afloat within the thermal puff, travel farther than the rest of the diameters. The intermediate size of 64 *μ*m, large enough for the fall outside the thermal puff relatively early but too small to retain the injection momentum, exhibits the smallest horizontal spread.

**FIG. 7.**
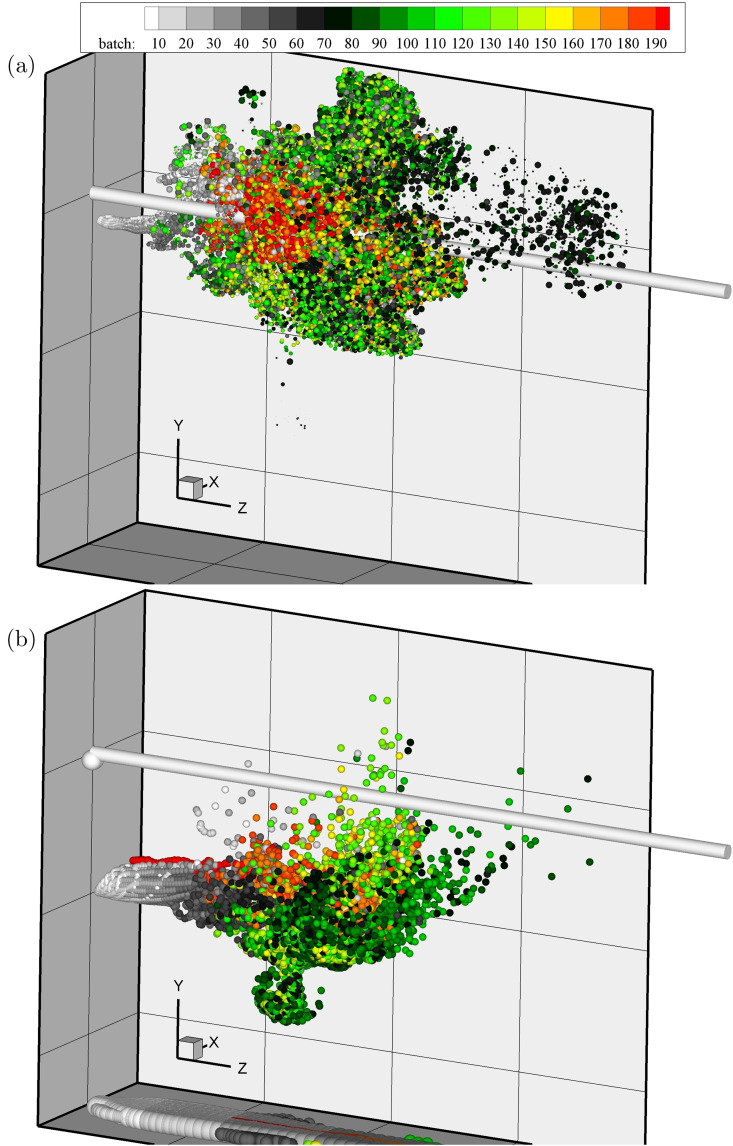
Snapshot of the particle position at the latest time t≈1.68 s. Markers are scaled by particle size and colored by the batch index. (a) 4–32 *μ*m and (b) 64–256 *μ*m. The projected inlet pipe in *z* is shown as a gray cylinder.

The 4–32 *μ*m and 64–256 *μ*m particle instantaneous positions for the nonevaporative case are shown in Figs. 7(a) and 7(b), respectively. Markers are scaled according to particle size and colored by the batch number with the first batch released at *t* = 0 and the last one *n_b_* = 200 at approximately *t* = 0.4 s. For reference, the inlet section has been projected as a cylinder spanning over the entire domain. The frontal puff region in Fig. 7(a) is characterized by the presence of particles released at the peak injection velocity (batch 70 in black). While particles released at early times during the initial stages of the cough (for instance, in white) tend to fall due to gravitational effects, those injected with similar velocity at the end of the expiratory event (in red) remain afloat dragged by the growing puff.

In the case of large particles shown in Fig. 7(b), both whitish and reddish particles capable of rapidly falling outside the puff due to gravity exhibit similar vertical position distributions. Both 128 and 256 *μ*m appear at the bottom of the computational domain after describing quasi-parabolic trajectories.

**FIG. 8.**
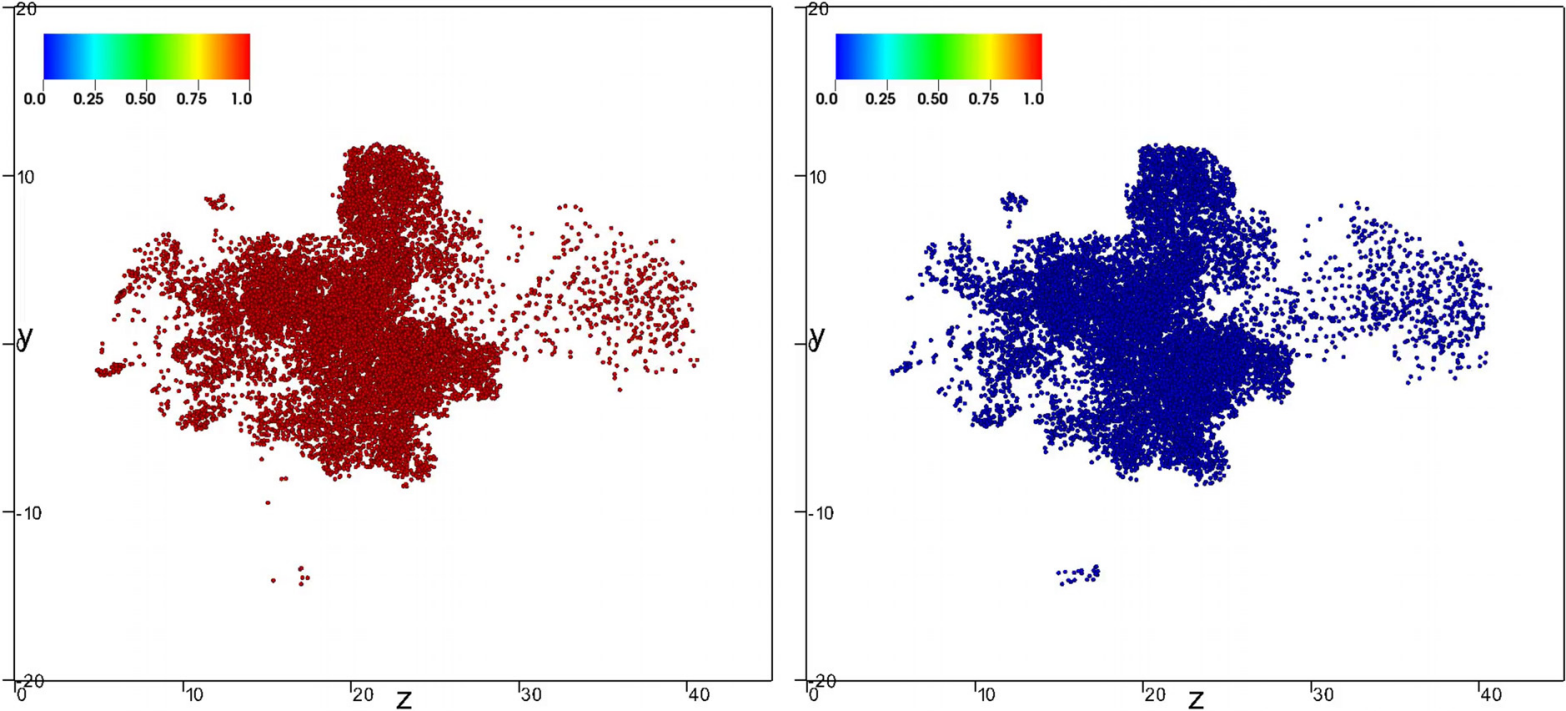
Lateral view of the position for both evaporative (right panel) and nonevaporative (left panel) particles with diameter 8 *μ*m at the latest time t≈1.68 s. The marker color corresponds to the value of *ς* defined in Eq. (5). Multimedia view: http://dx.doi.org/10.1063/5.0045416.1
10.1063/5.0045416.1

**FIG. 9.**
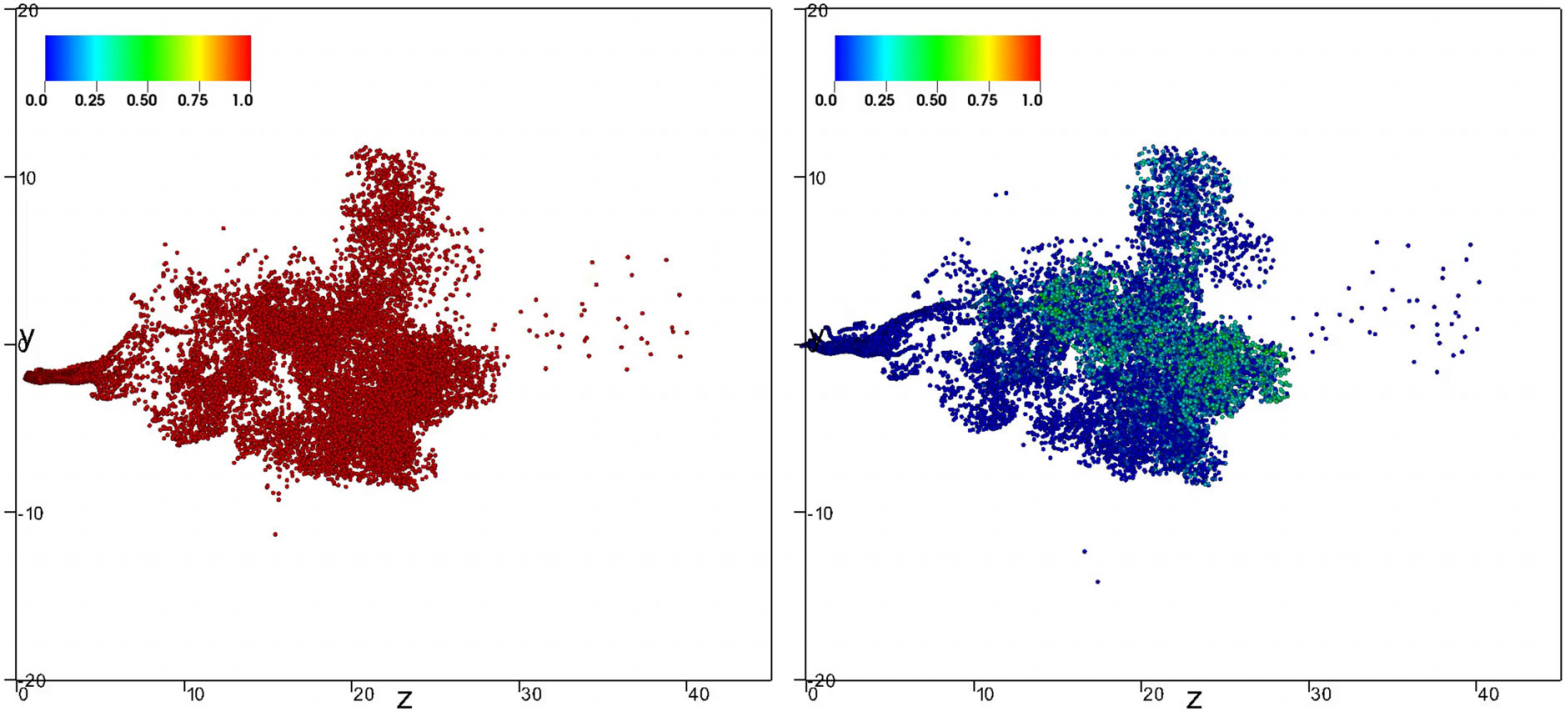
Lateral view of the position for both evaporative (right panel) and nonevaporative (left panel) particles with diameter 32 *μ*m at the latest time t≈1.68 s. The marker color corresponds to the value of *ς* defined in Eq. (5). Multimedia view: http://dx.doi.org/10.1063/5.0045416.2
10.1063/5.0045416.2

**FIG. 10.**
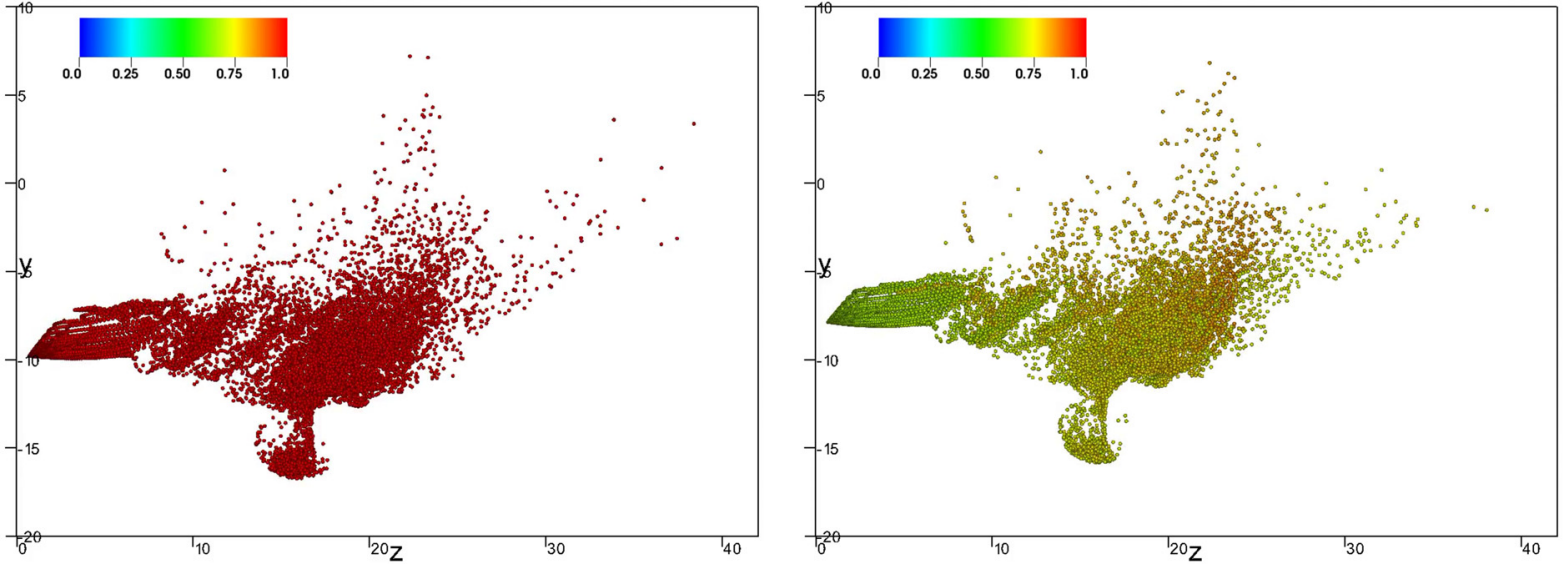
Lateral view of the position for both evaporative (right panel) and nonevaporative (left panel) particles with diameter 64 *μ*m at the latest time t≈1.68 s. The marker color corresponds to the value of *ς* defined in Eq. (5). Multimedia view: http://dx.doi.org/10.1063/5.0045416.3
10.1063/5.0045416.3

Figures 8, 9, and 10 (Multimedia view) show the of nondimensional particle position at the latest time t≈1.68 s for both evaporative (right panel) and nonevaporative (left panel) particles of initial diameters 8, 32, and 64 *μ*m, respectively. The marker color corresponds to the value of *ς* defined in Eq. (5).

**FIG. 11.**
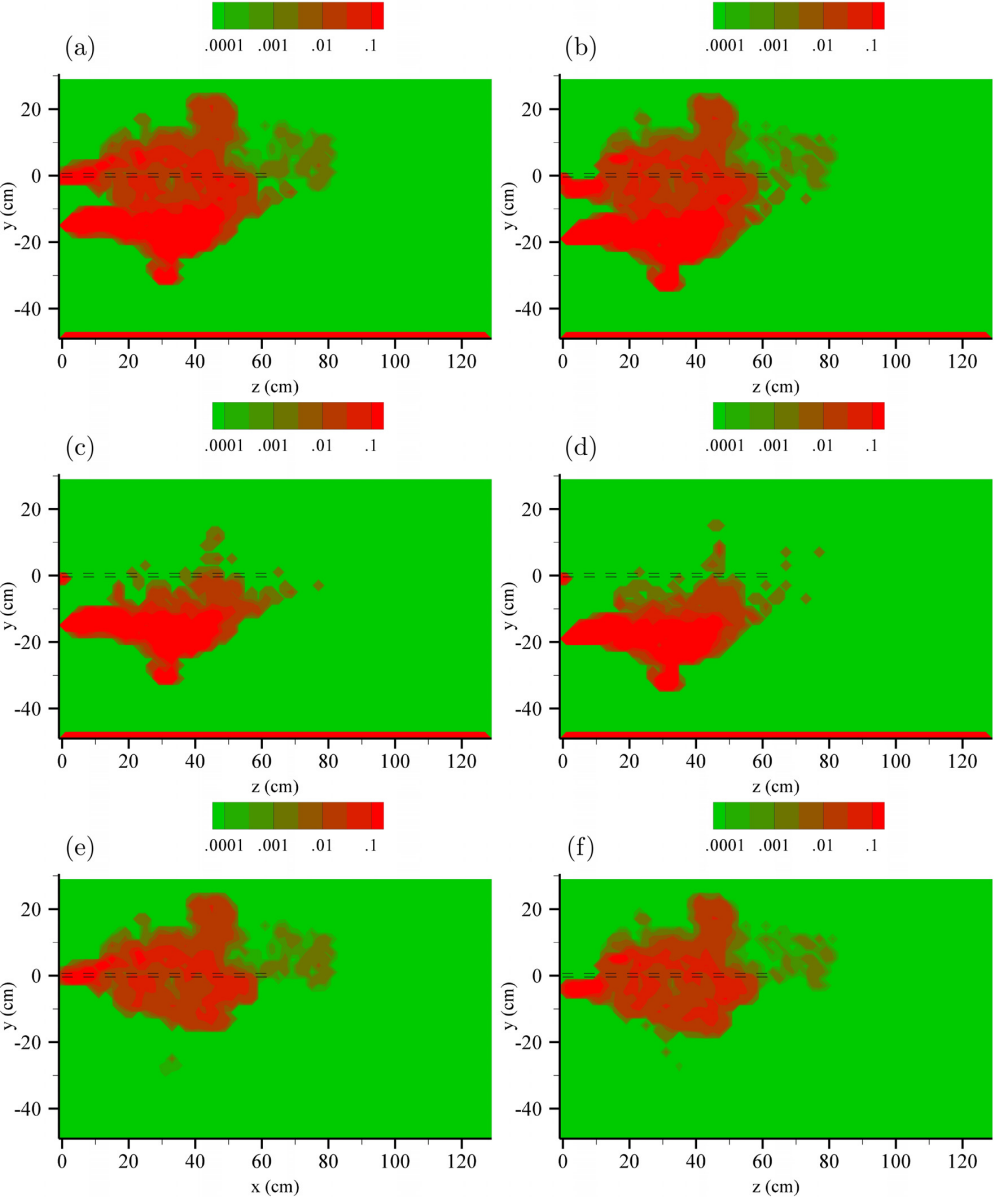
x = 0 slice of potential viral concentration *ζ* in nl of ejecta fluid × 1000 emitted particles per cm^3^ of air. (a), (c), and (e) Evaporative particles. (b), (d), and (f) Nonevaporative particles. (a) and (b) All particles. (c) and (d) 4–32 *μ*m. (e) and (f) 64–256 *μ*m. The projected inlet pipe in *z* is shown as black dashed lines.

We computed the potential viral concentration in the air using the positions of the particles at the maximum time reached by the simulation (t=1.68 s). As indicated above, at this time, the momentum of the puff has decreased to velocity values typical of indoor conditions. We have counted the particles in bins of 2×2×2 cm^3^, and the potential viral concentration has been calculated as the initial volume of the expelled droplet (nl) in a given bin multiplied by the part per thousand of the droplets of a given diameter in the bin, according to a typical droplet size distribution of expiratory events reported by Duguid,^50^ and divided by the volume of the bin (8 cm^3^). The potential viral concentration nl/1000 cm^3^ in the air resulting from the injection of 1000 ejected particles is shown in Fig. 11 for the evaporative (left panels) and nonevaporative (right panels) cases for all particles (top row), 64–256 *μ*m (central row) and 4–32 *μ*m (bottom row). The total viral load per air volume can be computed multiplying the potential viral concentration by the number of droplets expelled divided by 1000 and by the concentration of virus per volume of expelled material. The oral fluid average virus RNA load is 7 copies per nl with maximum up to $2.35\times 10^{3}$ copies per nl.^52^ For COVID-19, the minimum infectious dose is unknown, but it is estimated to be around a hundred virus particles.^61^ Considering that one cough releases around 5000 droplets,^50^ a value of the potential viral concentration of 0.01 corresponds to 350 copies per liter of air. The volume of inhaled air during normal breathing is about 0.5 l, and consequently, the inhalation of the air in the regions marked in red in Fig. 11 will produce at least the intake of 175 virus copies per inhalation.

As suggested by the results in Fig. 5, the overall effect due to evaporation is limited. Despite the larger horizontal range of the particle cloud, the low particle concentration in the puff front results in a reduced potential viral concentration in that region. Nonzero potential viral concentration levels exclusively due to large particles are mostly found at the location lower than the source (dashed horizontal black lines). On the other hand, small particles are responsible for vertically wider risk maps due to their capacity to rise above the source height level.

Overall, for the conditions considered in this simulation, the horizontal range of the viral concentration cloud before typical ambient air currents will likely dominate the aerosol dispersion is around 80 cm from the emitter.

## IV. CONCLUSIONS

Characterized by its transition from a laminar and then a turbulent jet into a buoyant puff, the numerical simulations of an idealized mild cough stressed the complex dynamics of the flow produced by expiratory events. Equipped with the fully resolved hydrodynamics and using a transport model for inertial particles, the results presented here demonstrate the impact of the background flow on the dispersion and fate of airborne droplets and aerosols produced by the breakup of ejected mucosa in rapid air exhalations.

Due to the significant impact of the particle size on the ability of the background flow on transporting it, the disperse phase model accounts for diameter reduction due to evaporative effects. Thus, droplets and aerosols produced by a cough are simulated by continuously seeding idealized spherical particles of different initial diameters and evaporation rates over the duration of the expiratory event. Individual trajectories are combined to obtain cloud statistics that allow us to elucidate the overall dispersion properties of the dispersed phase.

On the one hand, evaporation is found to be relevant only for a certain range of particle diameters for which the size reduction due to water content loss produces a transition of the dominant transport contribution from gravity to drag-dominated regimes over the span of the particle excursion.

We show that the cloud dynamics of aerosols under 64 *μ*m is characterized by an upward vertical drift produced by the buoyant puff. In contrast, larger particles tend to settle due to gravitational effects. Combining this detailed particle dispersion information with several reasonable assumptions on the viral load of typical ejecta fluid, we derived a viral concentration map for idealized environments that can be used to assess the infection risk under typical ambient conditions. In the future, the techniques used in the current study will be extended to other expiration events, e.g., talking, which has been postulated to be the primary mechanism for asymptomatic transmission. In the future, the importance of evaporation on long-term Heating, Ventilation, and Air Conditioning (HVAC) induced dispersion of aerosols is also a topic that will be explored.

## Data Availability

The data that support the findings of this study are available from the corresponding author upon reasonable request.
